# Language of primary medical qualification and differential MRCGP exam attainment: an observational study

**DOI:** 10.3399/BJGP.2024.0296

**Published:** 2025-02-25

**Authors:** Victoria Tzortziou Brown, Joanne Haviland, Garima Priyadarshini, Melody Turner, Riya Elizabeth George, Aloysius Niroshan Siriwardena, Simon Gregory

**Affiliations:** Wolfson Institute of Population Health, Queen Mary University of London, London.; Wolfson Institute of Population Health, Queen Mary University of London, London.; Wolfson Institute of Population Health, Queen Mary University of London, London.; Great Ormond Street Institute of Child Health, University College London, London; visiting researcher, The Healthcare Improvement Studies Institute, University of Cambridge, Cambridge.; Faculty of Medicine and Dentistry, Queen Mary University of London, London.; University of Lincoln, Lincoln.; Homerton College, University of Cambridge, Cambridge.

**Keywords:** differential attainment, educational assessment, general practice, language training, medical education

## Abstract

**Background:**

International medical graduates (IMGs) comprise more than half of GP registrars but are more likely to fail postgraduate assessments than UK graduates.

**Aim:**

To assess whether there is an association between the language of the primary medical qualification (PMQ) and Membership of the Royal College of General Practitioners (MRCGP) results, and whether performance in previous prequalification assessments is correlated.

**Design and setting:**

Retrospective observational study in the UK.

**Method:**

The World Directory of Medical Schools and the UK Medical Education databases were used to obtain data for all candidates who sat the MRCGP exams between October 2013 and July 2021 (*n* = 28 020). Candidates were split into three cohorts: cohort 1 comprised UK graduates; cohort 2 comprised IMGs with English as the language of the PMQ, who trained in countries with English (cohort 2a) or non-English (cohort 2b) as a first language; and cohort 3 included IMGs with non-English as the language of the PMQ. Logistic and linear regression analyses were used to compare the odds of exam passing and the scores relative to pass. Associations with past Multi-Specialty Recruitment Assessment (MSRA) scores, International English Language Testing System (IELTS) scores, and Professional and Linguistic Assessments Board (PLAB) scores were examined.

**Results:**

IMGs who trained in countries with non-English as a first language had statistically significantly lower odds of passing the exams and lower exam scores across all exam components. There were statistically significant positive correlations between MRCGP exam scores and MSRA, IELTS, and PLAB scores.

**Conclusion:**

English being the language of the PMQ and undertaking medical training in a country with English as the native language seemed to result in statistically significantly better chances of passing the exams and better exam scores. Performance in prequalification assessments can help to identify those IMG registrars who may benefit from tailored support.

## Introduction

The UK GP workforce is growing, with a large increase in international medical graduates (IMGs) whose primary medical qualification (PMQ) is gained outside of the UK. It is estimated that more than half (52%) of GP registrars are IMGs.^1^ The Membership of the Royal College of General Practitioners (MRCGP) examination is the postgraduate medical qualification currently required to attain a certificate of completion of training in general practice and, therefore, practise independently as a GP. The MRCGP examination comprises:

- one formative component (workplace-based assessment); and
- two summative components:
  — the Applied Knowledge Test (AKT), which is a computer-based assessment testing the knowledge base behind independent general practice in the UK in the context of the NHS; and
  — a consultation assessment testing the candidate’s ability to integrate and apply clinical, professional, and communication skills. Prior to 2020, this used an objective structured clinical exam (OSCE) format and was called the Clinical Skills Assessment (CSA); between 2020 and 2023 it used prerecorded video or audio consultations and was called the Recorded Consultation Assessment (RCA).^2^

IMGs have a lower pass rate in MRCGP exams than UK graduates (UKGs).^3^ According to the latest MRCGP annual report covering 2022/2023, the AKT pass rate for UKGs was 85%, whereas it was 56% for IMGs; the RCA pass rate has been reported as being 94% for UKGs and 57% for IMGs.^4^ The reasons for this are not fully understood. Candidates sitting the MRCGP are all UK-based GP specialty registrars, but their PMQ may be from one of 81 different countries.^3^ Analysis of MRCGP data has shown that the country of PMQ (UK or international) is a strong predictor of scores and pass/fail outcomes in all exam components.^5^ The impact of ethnicity and sex on exam attainment has been assessed in several studies and these demographic characteristics are frequently mentioned in exam annual reports;^3^^,^^5^^–^8 the impact of other factors — such as age, socioeconomic status, disability, language, and nationality — is less well understood and relevant data can be challenging to obtain.

**Table:** How this fits in

To the authors’ knowledge, this is the first study on the association between the language of the primary medical qualification and attainment in the Membership of the Royal College of General Practitioners (MRCGP) examination. It shows that undertaking undergraduate clinical training in a country where the native language is not English can statistically significantly and negatively affect examination performance in MRCGP exams. The study also shows statistically significant positive correlations between Multi-Specialty Recruitment Assessment, International English Language Testing System, and Professional and Linguistic Assessments Board scores and the MRCGP exam scores; this suggests that past performance in these assessments can help with the identification of those international medical graduate registrars who may find tailored support beneficial.

Qualitative studies have highlighted the importance of cultural and linguistic influences on clinical communication skills and, in particular, candidates’ ability to seek, detect, and acknowledge patients’ concerns in a high-stakes postgraduate clinical skills examination.^9^^,^^10^ Such cultural and linguistic influences can be different among a non-homogeneous group of IMGs that includes candidates who trained in medical schools in English and non-English language. The IMG group also includes an increasing number of British nationals who undertake their training outside of the UK. In 2021, an estimated 373 British nationals joined the UK medical workforce from universities in Bulgaria, Romania, the Czech Republic, Poland, and Hungary — all of which have English-language medicine programmes.^11^ Although there is evidence of the impact of PMQ place on exam attainment,^12^ it is unclear how PMQ language influences this. In addition, IMG candidates take other assessments — such as Multi-Specialty Recruitment Assessment (MSRA), International English Language Testing System (IELTS), and Professional and Linguistic Assessments Board (PLAB) tests — prior to entering their UK GP training programme. Previous research has shown that performance in such assessments can help to identify, at the outset of their training, those registrars who are likely to perform less well in the MRCGP;^12^^,^^13^ however, it is unclear how information on such performance can be used in relation to PMQ language.

Induction programmes have been established, with the aim of addressing some of the factors affecting the differential exam attainment observed for IMGs.^14^ A better understanding of the impact of PMQ language can assist towards the earlier identification of those registrars who would benefit from targeted support and can inform the design of examinations so that these can better reflect the competencies required when practising in an increasingly diverse society.

## Method

The authors combined the World Directory of Medical Schools (WDOMS) database, which contains information on PMQ language, and the UK Medical Education Database (UKMED), which collates data on the performance of UK medical students and registrars. Data were obtained for all candidates who sat the AKT and CSA or RCA components of the MRCGP exams between 30 October 2013 and 5 July 2021 (*n* = 28 020). The UKMED data dictionary^15^ provides a full list of data types, descriptions, and sources. For each of the three MRCGP exam components (AKT, CSA, and RCA) the authors used the D1_EXAM_PASSFAIL and D1_SCORE_RELATIVE_TO_PASS variables for first exam attempts. A combined variable representing overall pass/fail in AKT plus CSA/RCA exam was derived: overall pass was defined as a pass on both the AKT and either the CSA or RCA, whereas overall fail was defined as a fail on either the AKT or the CSA/RCA. As there is a varying pass mark for each exam, all candidates’ scores were scaled to a standard pass mark of zero for reporting purposes. These scores were reported as ‘score relative to pass’.

Three cohorts were defined as follows:

- cohort 1 — UKGs;
- cohort 2 — IMGs with PMQ English; and
- cohort 3 — IMGs with PMQ non-English.

Cohort 2 was split into two subcohorts:

- cohort 2a — this comprised IMGs with PMQ English, who trained in countries with English as a first and native language and who, therefore, undertook their clinical practice and patient interactions in English; and
- cohort 2b — this comprised IMGs with PMQ English, who trained in countries without English as a first and native language.

The primary aim was to assess for an association between PMQ language and passing the AKT, CSA, and RCA exam components. First, logistic regression analysis was used to compare pairs of cohorts in terms of the odds of passing the exams (overall and also for each individual component). Second, linear regression analysis was used to compare exam scores relative to pass between the cohorts. Statistical significance of the effect of cohort was assessed in the regression models using the Wald test.

The authors assessed whether confounders such as age, sex, ethnicity, and nationality influenced the associations of interest, and tested the effect of adjusting for these factors in the regression models. Socioeconomic status and learning disabilities were not adjusted for as cohorts 2 and 3 contained very limited data on these variables. The unadjusted and adjusted results were reported for age and sex, and for age, sex, ethnicity, and nationality. Similar regression analyses (logistic and linear) were also carried out to investigate whether there were associations between passing the AKT, CSA, and RCA exams and MSRA scores, IELTS scores, and PLAB scores for each cohort.

There were no missing data for age and gender, but ethnicity, nationality, and exam scores were unavailable for some candidates. Candidates with missing data were excluded from relevant analyses, but included in the sensitivity analyses using chained equations multiple imputation for missing data (with 10 iterations).

## Results

### Demographic characteristics

The cohort demographic characteristics are presented in Supplementary Table S1. There were more female candidates in cohorts 1 and 3 compared with cohort 2 (66% and 61% versus 53%, respectively). The mean age at first attempt of the exam was 31 years for cohort 1, 37 years for cohort 2, and 38 years for cohort 3. There were differences in distributions of ethnicities between the cohorts, with a higher proportion of White candidates in cohort 1 (64%), a higher proportion of Black candidates in cohort 2 (31%), and a higher proportion of Asian candidates in cohort 3 (69%).

### Association between demographic characteristics and exam performance

The exam scores for all exam components were statistically significantly lower with older age at first attempt, particularly for the RCA exam (β = −1.58, 95% confidence interval [CI] = −1.67 to −1.49, *P*<0.001) (see Supplementary Table S2). The exam scores were higher for females versus males, with the effects, again, being greater for the RCA exam (β = 9.27, 95% CI = 8.16 to 10.39, *P*<0.001). The exam scores were statistically significantly lower for non-White versus White candidates, and for non-British versus British candidates for all exam components; the effects were greater for the RCA exam (β = −18.07, 95% CI = −19.10 to −17.03, *P*<0.001 and β = −21.52, 95% CI = −22.55 to −20.49, *P*<0.001, respectively) (see Supplementary Table S3). With the exception of the effect of nationality, these effects were consistent in each of the three cohorts, particularly for the AKT exam, in which non-British candidates scored higher than British candidates in cohorts 2 and 3.

### Association between PMQ language and exam performance

Both the odds of passing the exams and the exam scores relative to pass were statistically significantly lower for cohorts 2 and 3 compared with cohort 1 in unadjusted analyses, and also after adjusting for age and sex, and for age, sex, ethnicity, and nationality. Cohorts 2b and 3, which comprised candidates who did their clinical undergraduate practice (including patient interactions) in non-English language, had statistically significantly lower odds of passing the exams and lower exam scores compared with cohort 2a, which comprised candidates with PMQ English who did their clinical practice in English. This finding was observed across all exam components. The results are presented in Tables 1, 2, and 3. All results are first attempt.

**Table 1.** MRCGP exam passes and fails by cohort, with cohort 2 sub-divided into 2a (first language English) and 2b (first language not English)

**MRCGP exam**	**Cohort**				**Total, *N* (%)**
	**1, *n* (%)**	**2a, *n* (%)**	**2b, *n* (%)**	**3, *n* (%)**	
**Fail**	3835 (22)	115 (49)	3115 (79)	1020 (77)	8085 (35)
**Pass**	13 455 (78)	120 (51)	835 (21)	305 (23)	14 715 (65)
**Missing^a^**	4135	50	795	240	5220
**Total**	21 425 (100)	285 (100)	4745 (100)	1565 (100)	28 020 (100)

a *Percentages calculated excluding missing data. MRCGP = Membership of the Royal College of General Practitioners.*

**Table 2.** Cohort-wise comparison of odds of passing MRCGP exams at first attempt, with cohort 2 subdivided into cohorts 2a and 2b

**Exam**	**Cohort comparison**	**Unadjusted OR (95% CI)**	***P*-value**	**Adjusted^a^ OR (95% CI)**	***P*-value**	**Adjusted^b^ OR (95% CI)**	***P*-value**
**AKT and CSA/RCA^c^**	2a versus 1	0.29 (0.23 to 0.38)	<0.001	0.44 (0.33 to 0.58)	<0.001	0.64 (0.48 to 0.87)	0.004
	2b versus 1	0.08 (0.07 to 0.08)	<0.001	0.12 (0.11 to 0.13)	<0.001	0.26 (0.23 to 0.29)	<0.001
	3 versus 1	0.09 (0.07 to 0.09)	<0.001	0.15 (0.13 to 0.17)	<0.001	0.31 (0.26 to 0.37)	<0.001
	2b versus 2a	0.26 (0.19 to 0.34)	<0.001	0.27 (0.20 to 0.35)	<0.001	0.39 (0.29 to 0.54)	<0.001
	3 versus 2a	0.29 (0.22 to 0.39)	<0.001	0.33 (0.24 to 0.45)	<0.001	0.46 (0.33 to 0.64)	<0.001
	3 versus 2b	1.12 (0.96 to 1.29)	0.139	1.22 (1.05 to 1.43)	0.010	1.16 (0.97 to 1.37)	0.097

**AKT^d^**	2a versus 1	0.34 (0.26 to 0.44)	<0.001	0.48 (0.37 to 0.63)	<0.001	0.64 (0.48 to 0.86)	0.002
	2b versus 1	0.14 (0.13 to 0.15)	<0.001	0.21 (0.19 to 0.23)	<0.001	0.36 (0.32 to 0.39)	<0.001
	3 versus 1	0.15 (0.14 to 0.17)	<0.001	0.25 (0.22 to 0.29)	<0.001	0.44 (0.38 to 0.51)	<0.001
	2b versus 2a	0.42 (0.33 to 0.55)	<0.001	0.44 (0.34 to 0.58)	<0.001	0.55 (0.41 to 0.73)	<0.001
	3 versus 2a	0.45 (0.34 to 0.59)	<0.001	0.51 (0.38 to 0.68)	<0.001	0.63 (0.46 to 0.86)	0.004
	3 versus 2b	1.06 (0.94 to 1.20)	0.357	1.15 (1.02 to 1.31)	0.034	1.16 (1.04 to 1.34)	0.035

**CSA^e^**	2a versus 1	0.31 (0.22 to 0.44)	<0.001	0.45 (0.31 to 0.64)	<0.001	0.66 (0.45 to 0.98)	0.037
	2b versus 1	0.08 (0.08 to 0.09)	<0.001	0.13 (0.11 to 0.14)	<0.001	0.30 (0.26 to 0.35)	<0.001
	3 versus 1	0.09 (0.08 to 0.10)	<0.001	0.14 (0.12 to 0.16)	<0.001	0.35 (0.29 to 0.42)	<0.001
	2b versus 2a	0.27 (0.19 to 0.38)	<0.001	0.29 (0.20 to 0.42)	<0.001	0.41 (0.28 to 0.61)	<0.001
	3 versus 2a	0.29 (0.21 to 0.42)	<0.001	0.33 (0.23 to 0.49)	<0.001	0.47 (0.31 to 0.71)	<0.001
	3 versus 2b	1.09 (0.95 to 1.26)	0.188	1.15 (0.99 to 1.33)	0.062	1.15 (0.98 to 1.36)	0.093

**RCA^f^**	2a versus 1	0.23 (0.12 to 0.42)	<0.001	0.38 (0.19 to 0.74)	0.004	0.71 (0.34 to 1.46)	0.352
	2b versus 1	0.08 (0.07 to 0.09)	<0.001	0.12 (0.09 to 0.14)	<0.001	0.36 (0.28 to 0.46)	<0.001
	3 versus 1	0.06 (0.05 to 0.08)	<0.001	0.10 (0.08 to 0.14)	<0.001	0.26 (0.18 to 0.37)	<0.001
	2b versus 2a	0.34 (0.19 to 0.63)	<0.001	0.31 (0.16 to 0.59)	<0.001	0.44 (0.21 to 0.89)	0.023
	3 versus 2a	0.27 (0.14 to 0.52)	<0.001	0.27 (0.14 to 0.54)	<0.001	0.33 (0.15 to 0.69)	0.003
	3 versus 2b	0.79 (0.62 to 1.02)	0.071	0.88 (0.67 to 1.15)	0.354	0.75 (0.56 to 0.99)	0.048

a *Adjusted for age and sex.*

b *Adjusted for age, sex, ethnicity, and nationality.*

c n *= 22 795 (22 445 when adjusting for ethnicity and nationality).*

d n *= 25 315 (24 995 when adjusting for ethnicity and nationality).*

e *n = 19 555 (19 170 when adjusting for ethnicity and nationality).*

f n *= 4770 (4740 when adjusting for ethnicity and nationality). AKT = Applied Knowledge Test. CSA = Clinical Skills Assessment. MRCGP = Membership of the Royal College of General Practitioners. OR = odds ratio. RCA = Recorded Consultation Assessment.*

**Table 3.** Cohort-wise comparison of scores relative to pass for MRCGP exams at first attempt, with cohort 2 subdivided into cohorts 2a and 2b

**Exam**	**Cohort comparison**	**Unadjusted regression coefficient (95% CI)**	***P*-value**	**Adjusted^a^ regression coefficient (95% CI)**	***P*-value**	**Adjusted^b^ regression coefficient (95% CI)**	***P*-value**
**AKT^c^**	2a versus 1	−8.69 (−10.79 to −6.59)	<0.001	−5.33 (−7.40 to −3.25)	<0.001	−2.19 (−4.28 to −0.10)	0.040
	2b versus 1	−21.10 (−21.68 to −20.53)	<0.001	−16.89 (−17.51 to −16.27)	<0.001	−12.26 (−13.12 to −11.41)	<0.001
	3 versus 1	−19.89 (−20.87 to −18.89)	<0.001	−14.63 (−15.66 to −13.61)	<0.001	−9.68 (−10.87 to −8.49)	<0.001
	2b versus 2a	−12.41 (−14.85 to −9.97)	<0.001	−11.68 (−14.09 to −9.28)	<0.001	−9.83 (−12.53 to −7.14)	<0.001
	3 versus 2a	−11.19 (−13.79 to −8.58)	<0.001	−9.57 (−12.15 to −6.99)	<0.001	−7.56 (−10.40 to −4.72)	<0.001
	3 versus 2b	1.22 (−0.02 to 2.45)	0.054	2.08 (0.85 to 3.31)	0.001	2.29 (0.96 to 3.62)	0.001

**CSA^d^**	2a versus 1	−5.66 (−7.07 to −4.25)	<0.001	−3.89 (−5.26 to −2.52)	<0.001	−1.93 (−3.29 to −0.56)	0.006
	2b versus 1	−14.27 (−14.68 to 13.87)	<0.001	−11.82 (−12.25 to −11.39)	<0.001	−7.02 (−7.57 to −6.46)	<0.001
	3 versus 1	−13.78 (−14.35 to 13.21)	<0.001	−11.29 (−11.88 to −10.69)	<0.001	−6.39 (−7.09 to −5.69)	<0.001
	2b versus 2a	−8.62 (−10.13 to −7.10)	<0.001	−7.55 (−8.97 to −6.12)	<0.001	−4.76 (−6.32 to −3.19)	<0.001
	3 versus 2a	−8.12 (−9.69 to −6.55)	<0.001	−6.78 (−8.27 to −5.29)	<0.001	−4.00 (−5.62 to −2.39)	<0.001
	3 versus 2b	0.49 (−0.19 to 1.19)	0.161	0.77 (0.11 to 1.42)	0.022	0.67 (−0.06 to 1.39)	0.071

**RCA^e^**	2a versus 1	−13.69 (−17.92 to −9.46)	<0.001	−9.22 (−13.32 to −5.13)	<0.001	−5.25 (−9.29 to −1.19)	0.011
	2b versus 1	−24.92 (−25.97 to −23.86)	<0.001	−20.34 (−21.48 to −19.21)	<0.001	−10.47 (−12.04 to −8.90)	<0.001
	3 versus 1	−26.59 (−28.49 to −24.69)	<0.001	−20.84 (−22.82 to −18.87)	<0.001	−12.40 (−14.63 to −10.18)	<0.001
	2b versus 2a	−11.22 (−15.58 to −6.87)	<0.001	−11.08 (−15.18 to −6.98)	<0.001	−5.81 (−10.36 to −1.26)	0.012
	3 versus 2a	−12.90 (−17.54 to −8.26)	<0.001	−11.20 (−15.59 to −6.81)	<0.001	−7.27 (−12.07 to −2.48)	0.003
	3 versus 2b	−1.68 (−3.72 to 2.37)	0.108	−0.08 (−2.05 to 1.89)	0.937	−1.31 (−3.38 to 0.76)	0.215

a *Adjusted for age and sex.*

b *Adjusted for age, sex, ethnicity, and nationality.*

c n *= 25 315 (24 995 when adjusting for ethnicity and nationality).*

d n *= 19 555 (19 170 when adjusting for ethnicity and nationality).*

e n *= 4770 (4740 when adjusting for ethnicity and nationality). AKT = Applied Knowledge Test. CSA = Clinical Skills Assessment. MRCGP = Membership of the Royal College of General Practitioners. OR = odds ratio. RCA = Recorded Consultation Assessment.*

### Association between MRCGP exam scores and MSRA, IELTS, and PLAB scores

The MSRA scores were statistically significantly lower for cohorts 2 and 3 compared with cohort 1 (see Supplementary Table S4). Statistically significant positive correlations were found between first-attempt MSRA scores and each component of the MRCGP exam for all cohorts together and each cohort separately, except for the RCA exam for cohort 3 (see Supplementary Table S5). MSRA scores had a stronger correlation with AKT exam scores and for cohort 1 (β = 0.67, *P*<0.001).

Positive correlations were found between IELTS overall scores and the scores relative to pass for each component of the exam for cohort 2 (see Supplementary Table S6) and cohort 3 (see Supplementary Table S7). Higher listening IELTS scores were associated with higher AKT, CSA, and RCA scores. Higher IELTS reading and writing scores were associated with higher AKT and CSA scores. Higher IELTS speaking scores were associated with higher CSA and RCA scores.

PLAB part 1 (PLAB 1) and PLAB part 2 (PLAB 2) scores were statistically significantly lower for cohort 3 compared with cohort 2 (see Supplementary Table S8). There were statistically significant positive correlations between PLAB scores and each component of the MRCGP exam for cohorts 2 and 3 (see Supplementary Table S9). Regression coefficients for PLAB1 were larger for AKT than CSA and RCA for cohort 2 (AKT unadjusted β = 0.38, *P*<0.001; CSA unadjusted β = 0.09, *P*<0.001; and RCA unadjusted β = 0.08, *P* = 0.066) and cohort 3 (AKT unadjusted β = 0.33, *P*<0.001; CSA unadjusted β = 0.08, *P*<0.001; and RCA unadjusted β = 0.10, *P* = 0.054); this indicates that higher PLAB1 scores were associated with higher scores relative to pass on AKT, but less so for the CSA/RCA. For PLAB2, regression coefficients indicated that higher scores were associated with higher scores for all components of the MRCGP exams, but this was particularly the case for the CSA (unadjusted β = 0.78, *P*<0.001 for both cohorts).

### Sensitivity analysis

The sensitivity analysis showed similar results to the unimputed analysis. The results of the analysis using imputation to assess the association between the odds of passing the exams and exam scores with PMQ language are presented in Supplementary Tables S10 and S11.

## Discussion

### Summary

The results showed that IMGs who trained in countries with non-English as a first language had statistically significantly lower odds of passing the exams and lower exam scores across all exam components. There were statistically significant positive correlations between MRCGP exam scores and MSRA, IELTS, and PLAB scores.

### Strengths and limitations

To the authors’ knowledge, this is the first study combining two datasets (WDOMS database and UKMED) to assess the association between PMQ language and MRCGP examination attainment. It showed that undertaking undergraduate clinical training in a country in which the native language is not English can statistically significantly and negatively affect examination performance in both the AKT and the CSA/RCA exam components. It also explored the effect of age and nationality, which are important confounders in exam performance.

In the analysis, only performance at first attempt was considered. GP registrars are allowed up to a maximum of four attempts of the AKT and the CSA/RCA components, and it could be argued that the first attempt in these examinations may not be a true reflection of their overall performance. Subsequent scores may be higher, but only those who fail their first attempt have more than one score. It has, however, been shown that the mark at the first attempt of taking an examination is a good predictor of future performance;^16^ therefore, first-attempt data were used for the analysis, as has been the case with previous research.^12^^,^^16^

The authors were unable to undertake a more-detailed analysis of the effects of ethnicity on examination performance by breaking down ethnicity to smaller subgroups because the numbers of candidates in cohorts 2 and 3 were too small. Furthermore, the authors were unable to consider the effect of other factors, such as disability and socioeconomic status, on examination performance due to data limitations.

### Comparison with existing literature

The results show that IMG cohorts with different PMQ languages can be very different demographically in terms of ethnicity, sex, and age. The analysis showed that the odds of passing and exam scores were statistically significantly higher with younger age at first attempt, for females versus males, for White versus non-White candidates, and for British versus non-British candidates (for all MRCGP exam components). These effects were consistent in each of the three cohorts, apart from the effect of nationality, particularly for the AKT exam, in which non-British candidates scored higher than British candidates in cohorts 2 and 3. Although the numbers were small and the 95% CIs were wide, this finding may reflect different undergraduate performance of British candidates choosing to undertake their medical training outside of the UK (cohort 2) or linguistic barriers of British candidates undertaking their training in another language (cohort 3).

The study presented here did not identify statistically significant differences in exam attainment between cohorts 2 and 3, demonstrating that PMQ language on its own does not seem to influence exam performance. However, the subgroup analysis showed that English PMQ language and undertaking clinical undergraduate practice (including patient interactions) in English seemed to result in statistically significantly better chances of passing the exams and better exam scores relative to pass across all exam components compared with those achieved by candidates who trained in countries without English as a first language. These results are consistent with studies that emphasised the importance of cultural and linguistic factors on CSA exam performance, as outlined below.

Hymes^17^ lists sociolinguistic competence (using language appropriate to context) and grammatical competence (language proficiency) as two factors in communicative competence. Understanding colloquial language or ‘patient speak’ and being aware of the subtleties of UK standard English — including the rhythm and intonation of speech that conveys both information and attitudes, and the non-verbal components of communication — can be important for successful interactions with patients.^18^^,^^19^ Research on the CSA assessment showed that the relatively decontextualised nature of the exam makes it a ‘talk-heavy’ assessment, in which communicative performance factors contribute to the gap in success rates between IMGs and other candidates.^20^ The higher rate of failure in this group has been shown to partly relate to some lack of clarity in all types of explanations to patients, higher rates of misunderstandings, and higher rates of candidates being perceived by examiners to use formulaic language and being unengaging.^20^ Being taught medicine and undertaking undergraduate clinical practice in another language seems to be an important influencing factor for exam attainment that has not been sufficiently explored so far. The place of PMQ, which has been accounted for in Patterson *et al*,^12^ does not necessarily reflect the language used to teach: for instance, several medical schools in India teach in English whereas, in Brazil, Portuguese is commonly used in medical schools.

The effect on the AKT exam component, and not just on CSA/RCA, is of particular note. The finding that training in a country with a non-English first or native language affects written exam performance is consistent with evidence from other disciplines that shows that taking an exam in a second language leads to a loss in grade points.^21^^,^^22^ The reason could be due to the effects of switching language (including longer reaction times and lower accuracy), which apply to those situations in which the registrar has to mentally retrieve information that was previously acquired in a different language than the current exam language.^23^ Furthermore, there is evidence that foreign-language processing reduces the impact of intuition and/or increases the impact of deliberation on people’s choices,^24^ resulting in another possible source of cognitive costs when being examined in a different language.

There is evidence of a positive correlation between past educational achievement at secondary school and medical school and MRCP examination performance.^25^ The term ‘academic backbone’ has been used to describe the concept of developing sophisticated underlying structures of knowledge that provide the ‘cognitive capital’ and ‘medical capital’ that seem important for future medical exam performance.^25^ Research also shows strong correlations between MSRA scores and MRCGP exams.^12^ The results presented here were consistent with such evidence and showed statistically significant positive correlations between first-attempt MSRA scores and each component of the MRCGP exam for all cohorts together and each cohort separately (with the exception of the RCA exam in cohort 3). The closer correlation between AKT and MSRA scores could be because of their similar formats — both are computer-based assessments testing clinical knowledge.

In accordance with previous evidence,^12^ the authors of the study presented here found positive correlations between IELTS overall scores and the scores relative to pass for each component of the exam for both cohorts 2 and 3. Previous research showed that the listening component of IELTS was the best predictor for the CSA, whereas the reading component was the best predictor for the AKT exam.^12^ The study presented here showed that IELTS reading and writing scores could predict AKT performance, whereas speaking and listening IELTS scores could predict CSA and RCA performance.

In addition, and in agreement with previous evidence,^13^ the authors of the study presented here found a stronger association between PLAB1 scores and AKT exam scores, and between PLAB2 scores and CSA/RCA exam scores compared with other scores.

### Implications for research and practice

The study results highlight the need for an intersectional approach to be adopted — that is, accounting for age, sex, ethnicity, and nationality — when assessing differential exam performance in order to facilitate a better understanding of the influence of these factors. This does not happen consistently in relevant reports on examination outcomes at present. More-complete data collection on factors such as ethnicity, disability, and socioeconomic status of IMG candidates in the future may allow for these factors to be studied and accounted for when assessing differential exam attainment.

The lower PLAB scores analysis of cohort 3 compared with cohort 2 could be due to linguistic barriers. Differences were seen in both PLAB1 and PLAB2 examinations, but were greater for the PLAB2 exam — possibly because PLAB2 is an OSCE-type assessment that relies more heavily on good communication skills. Further research is required to explore this hypothesis.

The statistically significant positive correlations between MSRA, IELTS, and PLAB scores and AKT and CSA/RCA exam scores across all cohorts indicated that performance in these prequalification exams could help identify IMG registrars who may benefit from tailored support. The findings relating to the key language skills required, given the format of each exam, contributes to a better understanding of the theoretical underpinnings of differential attainment. As language skills are associated with MRCGP exam performance, IELTS scores may help towards the earlier identification of candidates who may need additional language support when embarking on their training.

The results are particularly relevant in view of the growing numbers and system reliance on IMG registrars in general practice. The insights generated may help the design of better tailored support through local and national schemes, such as the NHS Induction programme,^26^ which aims to welcome, value, and support all IMGs recruited to the NHS as they transition to UK clinical practice. The findings may also inform the development of medical assessments that more accurately test the required skills of registrars, while reflecting and valuing diversity. Consideration could be given to whether such assessments may need to move away from artificial exam time boundaries and closer to real-world case scenarios, with aspects of the consultation being assessed in the work environment as part of workplace-based assessments. Communicative alignment and flexibility, mutual understanding, multilingual communication, and undertaking consultations across language barriers are competencies that could also be assessed as they are becoming increasingly relevant in our diverse societies.

## Data

Source — UK Medical Education Database (UKMED) UKMEDP160 extract generated on 8 September 2022. Approved for publication on 6 October 2023. Victoria Tzortziou Brown is grateful to UKMED for the use of these data. However, UKMED bears no responsibility for their analysis or interpretation. The data include information derived from that collected by the Higher Education Statistics Agency Limited (HESA) and provided to the General Medical Council (HESA Data). Source: HESA Student Records 2002/2003 and 2014/2015. Copyright HESA. HESA makes no warranty as to the accuracy of the HESA Data, and cannot accept responsibility for any inferences or conclusions derived by third parties from data or other information supplied by it.
